# Combining independent *de novo* assemblies optimizes the coding transcriptome for nonconventional model eukaryotic organisms

**DOI:** 10.1186/s12859-016-1406-x

**Published:** 2016-12-09

**Authors:** Nicolas Cerveau, Daniel J. Jackson

**Affiliations:** Department of Geobiology, Goldschmidtstr.3, Georg-August University of Göttingen, 37077 Göttingen, Germany

**Keywords:** Transcriptome, De novo assembly, Eukaryote, Protein coding, Merge, Redundant

## Abstract

**Background:**

Next-generation sequencing (NGS) technologies are arguably the most revolutionary technical development to join the list of tools available to molecular biologists since PCR. For researchers working with nonconventional model organisms one major problem with the currently dominant NGS platform (Illumina) stems from the obligatory fragmentation of nucleic acid material that occurs prior to sequencing during library preparation. This step creates a significant bioinformatic challenge for accurate *de novo* assembly of novel transcriptome data. This challenge becomes apparent when a variety of modern assembly tools (of which there is no shortage) are applied to the same raw NGS dataset. With the same assembly parameters these tools can generate markedly different assembly outputs.

**Results:**

In this study we present an approach that generates an optimized consensus *de novo* assembly of eukaryotic coding transcriptomes. This approach does not represent a new assembler, rather it combines the outputs of a variety of established assembly packages, and removes redundancy via a series of clustering steps. We test and validate our approach using Illumina datasets from six phylogenetically diverse eukaryotes (three metazoans, two plants and a yeast) and two simulated datasets derived from metazoan reference genome annotations. All of these datasets were assembled using three currently popular assembly packages (CLC, Trinity and IDBA-tran). In addition, we experimentally demonstrate that transcripts unique to one particular assembly package are likely to be bioinformatic artefacts. For all eight datasets our pipeline generates more concise transcriptomes that in fact possess more unique annotatable protein domains than any of the three individual assemblers we employed. Another measure of assembly completeness (using the purpose built BUSCO databases) also confirmed that our approach yields more information.

**Conclusions:**

Our approach yields coding transcriptome assemblies that are more likely to be closer to biological reality than any of the three individual assembly packages we investigated. This approach (freely available as a simple perl script) will be of use to researchers working with species for which there is little or no reference data against which the assembly of a transcriptome can be performed.

**Electronic supplementary material:**

The online version of this article (doi:10.1186/s12859-016-1406-x) contains supplementary material, which is available to authorized users.

## Background

RNA-Seq is a flavour of NGS that can generate extremely powerful datasets for a variety of research themes. Gene discovery, digital gene expression profiling of entire tissues or developmental stages and population genetics [1, 2] are some of the broad applications to which this technology can be applied. For researchers working with nonconventional model organisms RNA-Seq is alluring because such analyses are often touted as being possible in the absence of an assembled genome to which such transcriptome data is ideally mapped. In these cases the researcher faces the significant bioinformatic challenge of accurately assembling an RNA-Seq dataset “*de novo”* [3]. This is a challenge because the currently dominant NGS platform (Illumina) requires nucleic acid samples to be fragmented prior to sequencing, a process that needs to be accurately bioinformatically reversed in order to reconstruct the original transcripts. Additionally, typical Illumina read lengths are much less than 500 bp long [4]. These features result in both genome guided and *de novo* transcriptome assembly approaches displaying a large number of bioinformatically derived artefacts, a phemonenon that is well known [3]. The challenge of generating an accurate assembly of a transcriptome has generated many responses from the scientific community [5–9], with each assembly package having its own strengths and weaknesses. One *de novo* assembly strategy has been to generate multiple assemblies with different k-mer values, to combine these and then remove the redundancy of the resulting merged assembly [10]. However this approach first requires the user to identify an appropriate range of k-mer values (not a trivial exercise), and may ultimately require the production of up to 62 transcriptomes for a single dataset [10]. Related to this issue is assessment of assembly quality. This issue is highlighted when one considers that different assembly packages applied to the same raw dataset usually generate markedly different outputs, even with the same assembly parameters, [10–12]. Any critical user would ask “which assembly is appropriate for my project?”. For datasets with high proportions of “novel” genes (often the case for nonconventional model organisms), this problem has few solutions that can be generally applied to all datasets. Statistics such as the N50, average transcript size or coverage are not usually informative nor relevant when assessing the quality of an RNA-Seq assembly [13]. Another approach is to focus on the annotatability of a given assembly. In combination with standard sequence similarity searches against public databases, the recently released BUSCO (Benchmarking Universal Single-Copy Orthologs) package falls under this umbrella, and can be used to assess the completeness of a given transcriptome or genome assembly [14].

Having been through the process of *de novo* transcriptome assembly optimization with our nonconventional model *Lymnaea stagnalis* (a freshwater pulmonate mollusc), we have developed a simple strategy that takes the consensus coding features of a set of three (or more) independent assembly packages, and discards redundancy. This is not a new assembly method, but a way to survey the outputs of different assembly packages in order to generate a transcritpome that aims to be closer to the biological truth. We test our approach on simulated reads derived from the reference genomes of a fly and a nematode, and also on previously analyzed and publicly available raw RNA-Seq data derived from four eukaryotic lineages: two plants, a yeast, a fly and a nematode. In addition, we analyzed new RNA-Seq data derived from our model organism, *Lymnaea stagnalis*. For each dataset, we performed *de novo* assemblies with three independently developed and widely used software packages (Trinity [15], CLC Genomics Workbench V8.5 and IDBA-tran V1.1.1 [16]). The outputs of these assemblies were then processed through our pipeline. We demonstrate both bioinformatically (using a range of annotation based comparisons) and by validation in the lab for the *L. stagnalis* data, that this approach does indeed capture the most ‘biologically correct’ set of transcripts.

## Methods

### Raw data acquisition

**Fig. 1:**
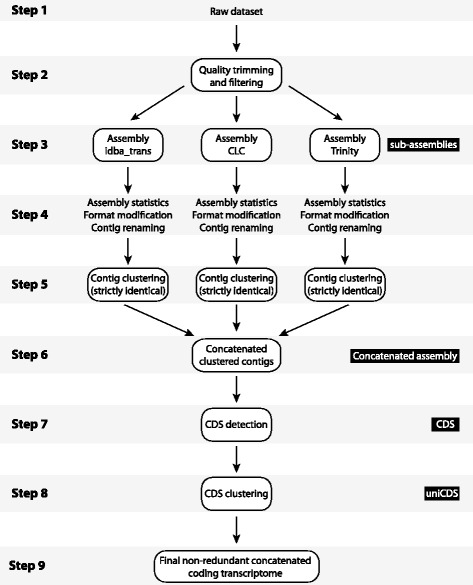
Schematic outline of our pipeline. Schematic representation of the steps involved in our pipeline

We used Illumina NGS data previously reported from three well-established model organisms: *Drosophila simulans*, *Caenorhabditis sp* and the unicellular eukaryote *Saccharomyces cerevisiae*. To increase the phylogenetic diversity of this selection we also included two plants, *Hippophae rhamnoides* and *Nicotiana benthamiana*. We also sampled the foot tissue of an individual *Lymnaea stagnalis* from our lab culture, and extracted total RNA following the protocol described in [17]. A stranded Truseq polyA library was constructed and paired end sequencing was performed on the Illumina HiSeq2000 platform. 46.5 million pairs raw reads were generated. 42.3 millon of these passed trimming and quality filtering and were used in all subsequent assembly analyses (Additional file 1: Table S1). The raw RNA-seq data for *Drosophila simulans*, *Caenorhabditis sp.*, *Saccharomyces cerevisiae*, *Hippophae rhamnoides* and *Nicotiana benthamiana* were obtained from the NCBI sequence read archive (SRA) (respective accession numbers: SRR1956911, ERR690851, SRR1924287, SRP011938 and SRA066161 (single end data omitted). The FASTQ data files were extracted using the SRA tool kit provided by NCBI. For all datasets, individual reads were quality filtered using Trimmomatic V0.32 [18] with the following parameters: LEADING:5 TRAILING:5 MINLEN:36 (step 2 in Fig. 1). For *L. stagnalis*, TruSeq primer sequences were clipped with the following parameter: ILLUMINACLIP:primer_file:2:30:10. The five datasets used in this study contained between 46,166,144 and 230,477,122 pairs of Illumina RNA-seq reads with read lengths of 100 bp, except for *S. cerevisiae* which had read lengths of 50 bp (Additional file 1: Table S1). Between 83 and 100% of the read pairs passed Trimmomatic quality checks (Additional file 1: Table S1). These quality filtered reads were used for our analyses.

### Generation of simulated Illumina reads from genomic references

We also generated artificial reads derived from the reference genomes of two well-established model organisms: *Drosophila melanogaster* and *Caenorhabditis elegans*. Genomic reference sequences and gff annotations were donwloaded from NCBI database for *D. melanogaster* (GCF_000001215.4_Release_6_plus_ISO1_MT) and *C. elegans* (GCF_000002985.6_WBcel235). Gff annotations were transformed into gtf format using the ‘rsem-gff3-to-gtf’ command from the Rsem package with the option mRNA for the RNA-pattern parameter [19]. Some annotations had to be deleted because strand information was not consistent with other records of the same transcript or CDS. The gtf files contained 30,421 transcripts for *D. melanogaster* and 28,014 for *C. elegans*. The *D. melanogaster* genome is composed of 1870 sequences and the *C. elegans* genome is composed of 7 sequences. Transcripts were extracted from *D. melanogaster* and *C. elegans* genomes using ‘rsem-prepare-reference’ from the Rsem package with the options mRNA for gff3-RNA-patterns and RefSeq for trusted-sources. Fifty Million read pairs were generated using the Flux simulator complete pipeline with simulated expression [20]. Library construction and sequencing simulation parameters for *D. melanogaster* are provided in Additional file 2. These artificially generated reads were also analyzed to calculate the read density per transcript. In order to represent variation in gene expression levels Flux simulator does not simulate reads on all input transcripts. We therefore removed transcripts that lacked simulated reads for all downstream analyses. As for Illumina datasets, simulated reads were quality filtered using Trimmomatic V0.32 [18] with the following parameters: LEADING:5 TRAILING:5 MINLEN:36 (step 2 in Fig. 1). In both datasets, 99.4% of the read pairs passed Trimmomatic quality checks (Additional file 1: Table S1). These reads were used for our analyses.

### Transcriptome assemblies

We selected three assembly packages with unique assembly strategies for our investigation: Trinity V2.0.3 [15], CLC Genomics Workbench V8.5 and IDBA-tran [16]. While all of these packages employ the *De Bruijn* method to perform their assemblies, CLC and Trinity use a single k-mer method whereas IDBA-tran uses a multiple k-mer method. In addition, while CLC and IDBA-tran produce a single *De Bruijn* graph (per k-mer for IDBA-tran and for the whole dataset for CLC), Trinity produces one *De Bruijn* graph per transcript, which are subsequently processed independently in order to extract all splice isoforms and to separate paralogous genes.

For each of the eight datasets we performed one assembly with each assembly package, resulting in three independent assemblies per dataset (step 3 in Fig. 1). CLC assemblies were run using a word of 20 base pairs (bp), a bubble size of 50 bp, with reads mapped back to the transcriptome using default parameters. IDBA_tran assemblies were run with k-mer values ranging from 20 to 100 bp with a step size of 10 bp. Trinity assemblies were run with default parameters and a k-mer value of 25. For each of these independent assemblies we recorded a variety of statistics (number of transcripts, smallest transcript, largest transcript, median transcript size, total assembly size, N50; step 4 in Fig. 1).

### Concatenated-assembly generation

Once the three individual assemblies for a given dataset had been generated we next produced a concatenated assembly. To do this we harmonized all assembly outputs into the same format. Transcript names were also modified so that the origin of each sequence in the concatenated-assembly could be traced (step 4 in Fig. 1). We then performed an intra-assembly clustering step in order to remove all strictly redundant transcripts present within each of the individual sub-assemblies for each dataset (step 5 in Fig. 1). For this clustering step we used CD-HIT-EST [21] with ten threads (-T), a maximum memory of 2549 megabytes (-M), local sequence identity (-G 0) with identity parameter of 100% (-c 1.00), minimal coverage ratio of the shorter sequence of 100% (-aS 1.00) and minimal coverage ratio of the longest sequence of 0.005% (-aL 0.005). The minimal ratio of the longest sequence was chosen in order to allow clustering of the whole range of transcript sizes. The resulting unique transcripts derived from each of the 3 assemblies for each dataset were then concatenated (step 6 in Fig. 1). TransDecoder V2.0.1 [22] was then used to detect open reading frames (ORFs) greater than 100 amino acids (step 7 in Fig. 1). The resulting coding sequence (CDS - i.e. with 5’ and 3’ UTRs removed), were then clustered again using CD-HIT-EST with minimal coverage ratio of the longest sequence of 0.005% (-aL 0.005), but a slightly lower sequence identity than the previous clustering step (-c 0.98) in order to take in consideration the Illumina sequencing error rate (step 8 in Fig. 1). The only parameter that can vary between clustering runs at this stage was the minimal coverage ratio of the shorter sequence (-aS). This parameter had values that ranged from 75 to 100% (100, 99, 98, 97, 96, 95, 90, 85, 80 and 75%). An -aS value was retrospectively selected in order to generate the most concise assembly (see below under *in silico* testing of assemblies). The resulting cluster info file (*.clstr) was retained in order to identify the transcript that generated the longest CDS of each cluster, and also all other transcripts of this cluster for further analyses. We mined the cluster information file to determine the assembly origins of each CDS and to calculate the CDS extension size (see below). The consensus of each cluster was then classified into one of seven categories:

1. The cluster consensus was present in all three assembler outputs
2. The cluster consensus was present in CLC and IDBA_tran outputs
3. The cluster consensus was present in CLC and Trinity outputs
4. The cluster consensus was present IDBA_tran and Trinity outputs
5. The cluster consensus was present only present in the CLC output
6. The cluster consensus was present only present in the IDBA_tran output
7. The cluster consensus was present only present in the Trinity output

The perl script (concatenator.pl) used to perform all of these steps is provided in the Additional file 3. As input this script requires the path to a directory containing the assembly outputs to concatenate, and the paths to two binary files: CD-HIT-EST and TransDecoder. Variable options include the nucleotide identity and the minimal coverage ratio of the shortest sequence for the CDS clustering step (step 8 in Fig. 1).

### Transcriptome assembly quality control

In order to test the quality of the assemblies generated by our pipeline we adopted two approaches, an annotatability based approach (applied to all datasets), and in vitro validation (applied to our *L. stagnalis* dataset).

#### Annotatability of assemblies

These analyses were performed with two different goals in mind. The first was to retrospectively determine the best minimal coverage ratio (aS value) for the final clustering step (in order to minimize redundancy and loss of information). To this end, we performed BLASTx searches for each of the above listed aS values, and BUSCO analyses for all assemblies based on Illumina datasets [14]. For BLASTx searches the e-value was set to 1e-3. A perl script was used to count the number of CDSs with a BLASTx hit. In addition, the number of unique BLASTx hits were counted. These values were compared across the different assemblies in order to identify at which aS value the concatenated-transcriptome began to lose information. The second goal was to evaluate any improvement that our concatenated-assembly approach gave relative to each of the individual assemblers. We applied Transdecoder to the transcripts generated by each individual assembler with the same parameters as described above. Subsequent BLASTx searches were also performed as described above for the concatenated-assembly. In addition, BUSCO analyses of individual and concatenated transcriptomes were also compared.

#### in vitro validation of the *L. stagnalis* assemblies

We performed a small scale in vitro validation of our new *L. stagnalis* transcriptome data using 10 randomly selected transcripts from each of the following categories outlined above: 1, 5, 6 and 7. Although this is a small sample compared to the overall transcriptome size, the resulting trends were informative. Transcripts were selected randomly using a perl script (Additional file 4). We designed primer pairs for each of these 40 selected transcripts with a melting temperature of 60 °C using Primer3 [23]. RT-PCR was performed on foot total RNA isolated from three *L. stagnalis* individuals (RNA derived from the individual used for NGS sequencing was not used in this exercise). Reverse transcription reactions were performed in a final volume of 25 μL as follows. One microgram of high quality total RNA was combined with 200 μmols of random hexamer and water to a final volume of 10 μL. This mix was put at 70 °C for 5 min in order to melt RNA secondary structure and allow primer annealing. The mix was then cooled to room temperature. We then added to each reaction Promega 5X MMLV-RT buffer (final concentration 1X), dNTPs (final concentration of 0.4 mM), 200 Units of MMLV-RT H^−^ mutant (Promega), and water to a final volume of 25 μL. For each reaction we performed a positive reverse transcription (RT+) containing all components mentioned above, and a negative reaction where MMLV-RT was replaced by water (RT-) to control for contaminating genomic DNA. Both RT+ and RT- reactions were incubated at room temperature for 10 min, and then heated to 42 °C for 90 min. The reactions were then heated to 70 °C for 15 min to inactivate the MMLV-RT. Single stranded cDNA was stored at -20 °C. PCR reactions were performed in a final volume of 25 μL containing the following: a final concentration of 1X enzyme reaction buffer, 0.2 mM dNTPs, 0.2 μM forward and reverse primers, 0.5 U Q5 polymerase (NEB), 1 μL of cDNA template and water to a final volume of 25 μL. Thermocycling was were performed in a SensoQuest thermocycler with the following steps: 94 °C for 10 min, 35 cycles with denaturation at 94 °C for 30 s, primer annealing at 55 °C for 30 s, DNA synthesis at 72 °C for 3 min with a final elongation step at 72 °C for 10 min. PCR products were loaded onto a 2% agarose gel containing ethidium bromide and electrophoresed at 130 V for 40 to 50 min and then visualized under UV light. For each primer pair, results were considered congruent when all three replicate RT+ reactions contained a distinct band at the expected size, and all three replicate RT- reactions were negative. A result was considered incongruent in any other case for the RT+ reactions. Reactions with negative and incongruent results were repeated a second time to confirm the results.

## Results and discussion

### Individual transcriptome assemblies

In order to broadly compare the outputs of the individual assemblers (CLC, Trinity and IDBA_tran) with our concatenated assemblies, we calculated some standard assembly metrics that are commonly used to characterize these kinds of datasets [13]. While each assembly output displayed different characteristics, a consistent pattern could be observed. Assemblies produced by Trinity always produced the highest numbers of transcripts and the largest transcriptome sizes (as measured by cumulating transcript lengths), whereas CLC generated assemblies with the lowest numbers of transcripts (except for the *S. cerevisiae* and *C. elegans* samples), and the smallest transcriptome sizes (Additional file 5: Table S2). Number of input reads did not have any influence on these metrics. Indeed, *S. cerevisiae* dataset has approximately twice the number of input reads than all other samples, and the smallest transcriptome output regardless of the assembly software. In general CLC and IDBA_tran produced 2.2 to 4.6 and 1.8 to 3.6 times fewer transcripts than Trinity respectively. N50 values for all assemblers lay between 405 and 4056 bp. IDBA_tran consistently generated the longest N50s, and CLC generally the smallest (except for the *S. cerevisiae*, *H. rhamnoides* and *C. elegans* datasets; Additional file 5: Table S2). However we must point out that the number of transcripts and N50 values will be biased by differences in the smallest transcript size assembled by each software (300 bp for IDBA-tran, 211 for CLC and 201 for Trinity; Additional file 5: Table S2), and also by the biological realities of these transcriptomes - longer N50 values do not necessarily reflect a better transcriptome assembly. The longest transcript sizes varied from 8609 to 51,362 bp, with the longest transcripts generated by Trinity (except for the *H. rhamnoides*, *N. benthamiana*, *D. melanogaster* and *C. elegans* datasets where it was generated by IDBA-tran). Interestingly, for some datasets the longest transcript size varied by more than two fold according to the assembler used (Additional file 5: Table S2). These general observations confirm previous reports that the use of different assemblers (even though they are all based on the construction of *de Bruijn* graphs), generate significantly different final assemblies [12, 24–26]. This led us to explore the possibility of combining these assemblies and removing any redundancy.

### Concatenated transcriptome assemblies

The main goal of our concatenated assembly approach was to improve assembly accuracy without generating a bloated assembly. In order to first remove intra-assembly redundancy, a stringent clustering step (100% sequence identity on 100% of the shorter sequence length) was performed for each individual sub-assembly (step 5 in Fig. 1). For all datasets, the redundancy rate was zero for all IDBA_tran assemblies and below 0.02% for all CLC assemblies (Additional file 6: Table S3). For Trinity transcriptomes, the redundancy rate was always significantly higher and ranged between 0.02 and 30% (Additional file 6: Table S3). The redundancy in the Trinity assemblies was also higher in the two simulated datasets (27 and 30%) than in the Illumina datasets (maximum 11%) (Additional file 6: Table S3). Higher intra-Trinity redundancy is probably due to the fact that Trinity is the only assembler to produce one *de Bruijn* graph per transcript, and subsequently processes them one by one, whereas CLC and IDBA_trans produce only one graph overall. The non-redundant transcripts produced by each assembler for each dataset were then pooled and TransDecoder was used to detect putative ORFs with a size of ≥100 amino acids. The resulting datasets had concatenated transcriptomes with 25,854 to 885,944 transcripts, and TransDecoder detected between 22,180 and 379,596 putative ORFs (Additional file 7: Table S4). Both simulated datasets fell within the range described by the Illumina datasets, while the proportion of the simulated transcriptomes predicted to be coding was higher (Additional file 7: Table S4). This is most probably due to the fact that simulated reads were derived from mRNA molecules.

The next critical step was to cluster the CDSs produced by Transdecoder in order to obtain the most concise coding transcriptome while minimizing information loss (step 8 in Fig. 1). To do this we used CD-HIT-EST with the nucleotide identity level set to 98% in order to be more conservative than the average Illumina sequencing error rate of 1%. The size ratio of the longest transcript to the overall transcript was set to 0.5% in order to include the shortest transcripts. The size ratio of the shortest transcript to the overall transcript (-aS) varied from 100 to 75% (see below). To evaluate the amount of information lost at this step, we used annotation-based metrics [13] that make more biological sense than metrics such as N50 or transcript size (however these can be found in Additional file 5: Table S2). BLASTx searches against Swiss-Prot database for each aS value were performed to determine the impact of the aS value in the above clustering step. It showed that the number of unique database entries decrease at 99% for both Illumina derived and simulated datasets (Additional file 8: Table S5). In addition, BUSCO analyses also showed that the completeness of each assembly began to decrease at an aS value of 99% for each sample. On the basis of these analyses (and to be the most conservative), the smallest aS value was set to 100% for all datasets. Nevertheless, it should be kept in mind that according to the dataset and the type of downstream analysis to be performed, a lower aS value may be more appropriate. After this clustering exercise, between 54 and 68% of the CDSs from each dataset were found to be redundant at the nucleotide level (Table 1). *C. elegans* dataset is in the range of the Illumina datasets whereas *D. melanogaster* is two point higher than the highest Illumina dataset, which is *D. simulans*.

**Table 1:** Assembly statistics

		Illumina derived datasets						Simulated datasets	
	Step^a^	*L. stagnalis*	*S. cerevisiae*	*Caenorhabditis sp.*	*D. simulans*	*H. rhamnoides*	*N. benthamiana*	*D. melanogaster*	*C. elegans*
Number of concatenated transcripts	6	576,412	25,854	152,491	184,892	278,987	885,944	42,535	15,340
CDS number	7	139,727	22,180	112,813	81,598	137,601	379,596	37,920	41,103
uniCDS number^b^	8	59,178 (58%)	9,942 (55%)	40,116 (64%)	27,735 (66%)	63,092 (54%)	131,656 (65%)	12,118 (68%)	14,890 (64%)
Total transcript number	9	58,185	9,744	39,022	26,968	61,798	127,526	11,582	14,283
Total CDS number	9	64,659	11,605	51,416	34,363	68,288	153,118	14,231	15,412
Transcripts with multiple CDSs^c^	9	5,759 (10%)	1,529 (15%)	9,756 (19%)	5,838 (22%)	5,999 (10%)	21,060 (17%)	2,218 (19%)	949 (7%)
Redundant CDSs^d^	9	5,481 (9%)	1,663 (14%)	11,300 (22%)	6,628 (19%)	5,196 (8%)	21,462 (14%)	2,113 (15%)	522 (3%)
Transcriptome size (bp)	9	131,591,076	16,164,888	69,689,679	69,421,322	86,181,833	206,036,224	34,121,269	19,765,122
Smallest transcript (bp)	9	300	300	300	300	300	300	300	300
Largest transcript (bp)	9	35,470	15,061	21,466	51,362	13,117	19,833	29,220	26,756
N50	9	3,483	2,414	2,366	3,866	1,823	2,116	4,479	1,666

^a^Step number in Fig. 1

^b^Proportion of discarded CDSs is indicated in brackets

^c^Proportion of transcripts with >1 CDS is indicated in brackets

^d^Proportion of none unique CDSs is indicated in brackets

Our concatenated coding transcriptomes ranged in size from 9744 transcripts for *S. cerevisiae* to 127,526 transcripts for *N. benthamiana* (Table 1). Whatever the raw data origin, the number of transcripts in the concatenated assembly is less than the number of uniCDSs, and the number of CDSs in the concatenated assembly is more than the number of uniCDSs (Table 1). This is because transcripts often possessed more than one CDS (Table 1). Transcripts with multiple CDSs also influenced the number of redundant CDSs in our concatenated assemblies (between 3 and 22%, Table 1). The proportion of CDS redundancy for the simulated *D. melanogaster* data is within the range of all Illumina datasets, while the simulated *C. elegans* is 5% lower than the smallest Illumina dataset (*H. rhamnoides* 8%). Transcripts with multiple CDSs may be the result of sequencing or assembly errors, the activity of transposable elements such as group-II intron or transposases that get inserted in genes [27], or operon transcription [28]. Compared to the individual assemblies generated by CLC, Trinity and IDBA-tran (Additional file 5: Table S2), the concatenated assemblies of *L. stagnalis*, *D. simulans* and *N. benthamiana* contained fewer transcripts than any of the individual sub-assemblies, whereas for *S. cerevisiae*, *Caenorhabditis sp*, *H. rhamnoides*, *D. melanogaster* and *C. elegans* the number of transcripts within the concatenated assemblies were within range of those produced by the individual assemblers. Considering the total transcriptome sizes, the concatenated assemblies were similar to the individual assemblies, but were always larger than the CLC generated assemblies (Table 1; Additional file 5: Table S2). For the *S. cerevisiae* dataset the concatenated transcriptome was larger than all individual assemblies (Table 1; Additional file 5: Table S2). Finally, the N50s of the concatenated assemblies were higher than all of the individual assemblies except for the *S. cerevisiae* and *C. elegans* dataset. This suggests that most of the transcripts removed during our concatenation and filtering steps had small sizes. These statistics also show that our pipeline did not increase the overall transcriptome size compared to the individual assemblers. In some cases the overall transcriptome size even decreased considerably (Table 1). This phenomena has also been previously observed in other plant datasets [6, 10]. The N50 values also suggests that our pipeline generates coding transcriptomes that have larger average transcript sizes than assemblies generated by the individual assemblers [13].

In order to further assess the performance of our pipeline, we compared transcripts generated by the three individual assemblers and our pipeline with the original transcripts from which artificial reads were generated for both the *D. melanogaster* and *C. elegans* datasets (Table 2). These comparisons were performed with BLASTn and we only considered hits with a nucleotide identity of 98% covering at least 50% of the original transcript. In both datasets, CLC failed to recover the highest proportion of genuine transcripts (21% for *D. melanogaster* and 34% for *C. elegans*), while our concatenated assemblies failed to recover the lowest proportion of genuine transcripts (11% for *D. melanogaster* and 29% for *C. elegans*). In general, these concerningly high values are similar to those previously made on human and worm *de novo* transcriptome assemblies [3]. For both the *D. melanogaster* and *C. elegans* datasets, most of the missing genuine transcripts in the concatenated assembly (85% for *D. melanogaster* and 80% for *C. elegans*) had read coverages of less than 10X, whereas most of the successfully recovered transcripts (82% for *D. melanogaster* and 89% for *C. elegans*) had read coverages higher than 10X.

**Table 2:** Comparison between original and assembled transcriptomes derived from simulated reads generated from *D. melanogaster* and *C. elegans* datasets

Organism	Assembler	Original transcripts		Assembled transcripts	
		Total number	Lacking a BLASTn hit in assembled transcripts	Total number	Lacking a BLASTn hit in original transcripts
*D. melanogaster*	Concatenated	11,856	1,277 (11%)	12,273	2,685 (22%)
	CLC		2,513 (21%)	8,113	1,972 (24%)
	IDBA_tran		1,775 (15%)	8,282	1,150 (14%)
	Trinity		1,843 (16%)	11,395	2,810 (25%)
*C. elegans*	Concatenated	16,513	4,774 (29%)	14,922	4,087 (27%)
	CLC		5,614 (34%)	11,853	2,948 (25%)
	IDBA_tran		5,018 (30%)	11,069	1,557 (14%)
	Trinity		5,473 (33%)	12,843	2,869 (22%)

Interestingly, and in contrast to the missing genuine transcripts, up to 27% of the assembled transcripts were not present in the original transcript set (representing bioinformatically ‘invented’ transcripts). IDBA_tran produces the lowest proportion of invented transcripts (14% for *D. melanogaster* and 14% for *C. elegans*), whereas Trinity produces the highest proportion of invented transcripts in *D. melanogaster* (25%) and CLC in *C. elegans* (25%). In *C. elegans*, our concatenated assembly had a higher proportion of invented transcripts than any single assembler, whereas in *D. melanogaster* it had a lower proportion than CLC and Trinity (Table 2).

### Evaluation of concatenated assemblies

**Fig. 2:**
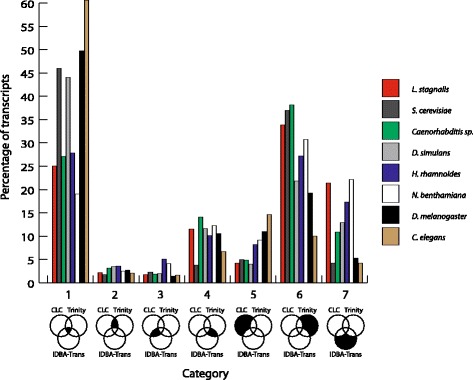
Categorization of concatenated clusters according to their presence/absence in the individual sub-assemblies. Category 1: clusters found in all three assemblers; category 2: clusters found in CLC and Trinity; category 3: clusters found in CLC and IDBA; category 4: clusters found in IDBA-tran and Trinity; category 5: clusters found in CLC; category 6: clusters found in Trinity and category 7: clusters found in IDBA

In order to study the composition of the final uniCDS clusters in our concatenated assemblies we assigned all clusters to one of seven categories (Fig. 2). The resulting pattern was consistent across all datasets, and all aS ratios used (75–100%) in the clustering step (data not showed). CDS clusters primarily belonged to either category 1 (the cluster was present in all three individual sub-assemblies following concatenation and redundancy filtering) or category 6 (the cluster was only present in the Trinity assembly) (Fig. 2). Of all three individual assemblers, Trinity consistently generated the most unique clusters (excepted for *C. elegans*), while CLC consistently generated the fewest unique clusters (excepted for *C. elegans*) (Fig. 2).

In order to compare these distributions between samples, we performed Kolmogorov-Smirnov statistical tests. All paired comparisons were statistically non-significant except for 4 that always involved at least one of the plant transcriptomes (one for *H. rhamnoides* and three for *N. bentamiana*) (Additional file 9: Table S6). Distribution comparisons between concatenated assemblies from both simulated datasets without plant Illumina datasets were always non-significant (Additional file 9: Table S6).

This categorization exercise led us to ask whether any one of these categories contained a higher proportion of “biologically correct” transcripts than others? In order to address this question, we performed an in vitro validation using the *L. stagnalis* dataset. We tested ten randomly selected clusters from categories 1 (clusters detected in all three assemblers), 5, 6 and 7 (clusters unique to either CLC, Trinity or IDBA_tran respectively). The positive validation rate for categories 5, 6 and 7 ranged from 40 to 70%, and the negative validation rate ranged from 30 to 60% (Table 3). Category 1 had a positive validation rate of 80%, and a negative validation rate of 0% (Table 3). These results suggest that clusters found by only one assembler (categories 5, 6 or 7) are likely to be either very lowly expressed or are assembly errors, while those found in all three assemblers (category 1) are more likely to be genuine molecules, giving further credence to the concept of our bioinformatic approach.

**Table 3:** In vitro validation of *L. stagnalis* clusters

	Number	Positive	Incongruent	Negative
Category 1 Clusters found in all 3 assemblers	10	8	2	0
Category 5 Clusters unique to the CLC assembly	10	2	5	3
Category 6 Clusters unique to the Trinity assembly	10	2	2	6
Category 7 Clusters unique to the IDBA_tran assembly	10	4	1	5

**Fig. 3:**
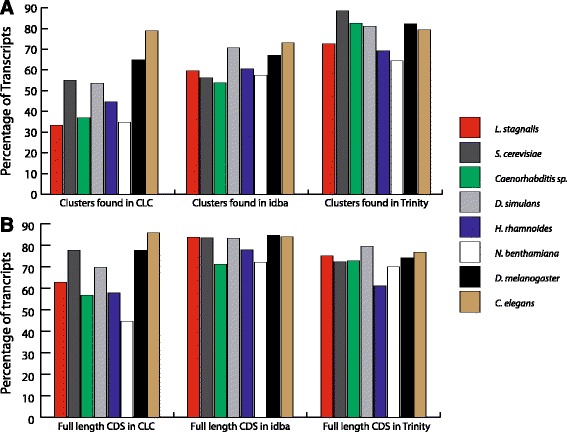
Characterisation of CDSs present in the final concatenated assembly and their presence/absence in the individual sub-assemblies. **a** Proportion of CDSs present in the final concatenated assembly present in each individual assembler for each dataset. **b** For those CDSs present in each sub-assembler (as in A), the proportion of CDSs from each individual assembler that matches (or exceeds) the length of the final concatenated CDS

We also retrospectively investigated the completeness of each individual assembly relative to our concatenated assemblies. The results of this analysis were striking. Averaging across all eight datasets, 50.3% ± 16.1% of CDS clusters in the concatenated assembly were present in the CLC assemblies, 62.3% ± 7.1% were present in the IDBA_tran assemblies and 77.5% ± 8.0% were present in the Trinity assemblies (Fig. 3a). Both simulated concatenated assemblies were in the range of all Illumina derived assemblies, excepted for CLC where the proportion of detected CDSs was higher than in any of the 6 other samples (Fig. 3a). On face value this result suggests that Trinity alone provides the best overall picture of a coding transcriptome. However, when we looked retrospectively at the effect of our pipeline on CDS extension, we found IDBA_tran to be the best performer for all datasets (except for both *Caenorhabditis* datasets; Fig. 3b). Between 13 and 44% of the CDSs from each assembler were extended during our concatenating process (Table 4). The proportion of extended CDSs from the simulated transcriptomes were within the range of all Illumina derived assemblies, excepted for CLC in *C. elegans* which was 3% lower than the smallest Illumina dataset (IDBA_tran in *S. cerevisiae*) (Table 4)*.*

**Table 4:** Effect of concatenating assemblies on CDS length

		Illumina derived datasets						Simulated datasets	
	Assembler	*L. stagnalis*	*S. cerevisiae*	*Caenorhabditis sp.*	*D. simulans*	*H. rhamnoides*	*N. benthamiaana*	*D. melanogaster*	*C.elegans*
Number of extended CDSs^a^	CLC	8,195 (38%)	1,333 (23%)	7,255 (44%)	5,490 (34%)	11,508 (38%)	24,218 (47%)	1,888 (23%)	1,534 (13%)
	IDBA_tran	13,401 (27%)	957 (16%)	11,717 (38%)	7,586 (25%)	11,427 (24%)	48,830 (36%)	1,960 (23%)	2,054 (18%)
	Trinity	23,532 (34%)	2,879 (27%)	28,918 (44%)	11,638 (33%)	21,032 (35%)	78,038 (40%)	7,817 (38%)	5,808 (33%)
Cumulated extended CDS length (bp)	CLC	9,289,434	913,872	6,758,496	5,921,460	6,816,978	23,238,501	2,662,092	1,107,756
	IDBA_tran	23,112,789	121,116	9,431,703	5,795,478	5,143,413	36,408,087	2,311,569	1,433,037
	Trinity	33,055,113	2,749,041	30,317,865	14,029,893	16,390,158	53,633,142	10,201,314	4,291,554
Mean extended CDS length (bp)	CLC	1,134	686	932	1,079	592	960	1,410	722
	IDBA_tran	1,725	127	805	764	450	746	1,179	698
	Trinity	1,405	955	1,048	1,206	779	687	1,305	739

^a^The proportion of CDSs with an extension are indicated in brackets

We also compared the annotatability of our concatenated transcriptomes relative to assemblies generated by each of the three individual assemblers using BLASTx sequence similarity searches against Swiss-Prot [13]. The results of these analyses showed that annotatability was always higher in the concatenated assemblies compared to all of the individual assemblies (Table 5). For all Illumina derived datasets, the proportion of CDSs with a BLASTx hit expressed as a percentage of that found in the corresponding concatenated assembly ranged between 94% for the Trinity assembly of the *S. cerevisiae* dataset to 36% for the CLC assembly of the *L. stagnalis* dataset (Table 5). This trend also held true for the *D. melanogaster* and *C. elegans* simulated datasets (Table 5).

**Table 5:** Comparisons of assembly annotatability

		Illumina derived datasets						Simulated datasets	
	Assembler	*L. stagnalis*	*S. cerevisiae*	*Caenorhabditis sp.*	*D. simulans*	*H. rhamnoides*	*N. benthamiana*	*D. melanogaster*	*C. elegans*
Number of uniCDS	Concatenated	59,178	9,942	40,116	27,735	63,092	131,656	12,118	14,890
	CLC	21,527	5,673	15,466	15,988	29,898	51,151	8,113	11,853
	IDBA_tran	36,726	5,608	21,384	19,556	38,353	79,612	8,271	11,069
	Trinity	44,545	9,339	34,356	23,509	46,571	88,428	11,351	12,838
Overall number of BLASTx hits	Concatenated	38,838	9,922	25,502	19,789	49,565	93,781	9,007	9,751
	CLC^a^	14,034	5,666	9,983	11,221	23,523	36,587	5,777	7,740
		(36%)	(57%)	(39%)	(57%)	(47%)	(39%)	(64%)	(79%)
	IDBA_tran^a^	23,634	5,598	13,996	14,107	30,491	56,665	6,218	7,489
		(61%)	(56%)	(55%)	(71%)	(62%)	(60%)	(69%)	(77%)
	Trinity^a^	30,134	9,320	21,730	16,765	36,232	63,079	8,342	8,559
		(78%)	(94%)	(85%)	(85%)	(73%)	(67%)	(93%)	(88%)
Number of unique BLASTx hits	Concatenated	15,232	5,404	9,242	9,575	15,524	16,700	4,957	5,767
	CLC^a^	10,958	5,094	7,492	8,376	12,405	12,902	4,664	5,529
		(72%)	94%)	(81%)	(87%)	(80%)	(77%)	(94%)	(96%)
	IDBA_tran^a^	12,893	5,223	8,069	8,868	13,840	14,900	4,655	5,302
		(85%)	(97%)	(87%)	(93%)	(89%)	(89%)	(94%)	(92%)
	Trinity^a^	14,124	5,243	9,051	9,174	14,314	14,855	4,817	5,340
		(93%)	(97%)	(98%)	(96%)	(92%)	(89%)	(97%)	(93%)

^a^Each value is also expressed as a percentage of the corresponding Concatenated dataset value (numbers in brackets)

We were aware that an increase in the proportion of CDSs returning a BLASTx hit does not necessary mean that annotation diversity also increases. Indeed, an overall increase in the number of BLASTx hits could be due to a greater number of mis-assembled isoforms or paralogs present in a given assembly. To account for this phenomenon we investigated annotation diversity by calculating the number of unique database entries for all BLASTx searches. Again in all cases the number of unique BLASTx hits was highest in the concatenated assemblies (Table 5). For the Illumina datasets, the number of unique database hits in the individual assemblies expressed as a percentage of that found in the corresponding concatenated assembly ranged between 98% (for the Trinity assembly of the *Caenorhabditis sp.* dataset) and 72% (for the CLC assembly of the *L. stagnalis* dataset; Table 5). These results demonstrate that an overall increase in the rate of annotation is accompanied by an increase in annotation diversity. This phenomena was also observed in the analysis of a *N. benthamiana* transcriptome [10]. It should be noted that the increase in annotation diversity in our concatenated assemblies was less extreme than the increase in the overall annotatability (Table 5). This implies that most of the increase in the overall annotation is due to CDS isoforms that were not found by a given individual assembler.

We also performed an analysis of assembly completeness using the transcription factor database BUSCO [14]. In addition to the simple presence/absence pattern of BUSCO entries, this analysis also provides interesting information regarding the number of duplicated and fragmented entries. The results of this analysis also confirmed the results obtained with our BLASTx searches; the number of detected BUSCOs entries was always higher in the concatenated assemblies than in all of the individual assemblers for all Illumina datasets and the simulated datasets (Table 6). In addition, the number of fragmented copies was always lower in all concatenated assemblies than in the individual sub-assemblies, except for the *Caenorhabditis* sp. dataset where the number of fragmented copies was equal in the concatenated and IDBA_tran assemblies and the *C. elegans* dataset where the number of fragmented copies is lower in Trinity and equal in IDBA_tran (Table 6).

**Table 6:** Results of BUSCO annotations

		*L. stagnalis*	*S. cerevisiae*	*Caenorhabditis sp.*	*D. simulans*	*H. rhamnoides*	*N. benthamiana*	*D. melanogaster*	*C. elegans*
		Illumina derived datasets						Simulated datasets	
BUSCO dataset		Metazoa	Fungi	Metazoa	Arthropods	Plants		Arthropods	Metazoa
Number of BUSCO entries		843	1,438	843	2,675	956		2,675	843
Detected BUSCO entries	Concatenated	822	1,357	720	2,455	903	934	1,204	442
	CLC	779	1,207	655	2,159	843	811	1,079	437
	IDBA_tran	813	1,195	699	2,242	881	903	1,137	428
	Trinity	811	1,356	710	2,424	887	927	1,143	415
Duplicated copies	Concatenated	344	430	445	945	525	745	368	49
	CLC	59	35	52	89	224	284	48	25
	IDBA_tran	210	70	189	662	361	569	149	28
	Trinity	259	155	324	520	389	633	291	42
Fragmented copies	Concatenated	19	53	16	143	20	5	89	54
	CLC	66	106	39	230	88	167	94	57
	IDBA_tran	20	59	16	174	33	63	93	54
	Trinity	35	136	24	189	52	8	100	53

There were always the fewest number of duplicated copies in all CLC sub-assemblies, but CLC was always the single assembler with the fewest total number of BUSCO entries, except for the *S. cerevisiae* and *C. elegans* datasets (Table 6). Our concatenated assemblies always contained a higher number of duplicated copies than all three individual assemblers. This is apparently a weakness of our methodology that must be traded off against an assembly with more copies and fewer fragmented copies (Table 6). Our concatenated assemblies produced from the simulated datasets reflected the same patterns seen in the Illumina derived data (Table 6).

Because the NCBI databases have evolved significantly over the last two years, we downloaded the previously reported [10] cumulative transcriptome of *N. benthamiana* (http://benthgenome.qut.edu.au/), repeated the BLASTx and BUSCO searches and compared these updated results to our assembly of the same raw data. This comparison revealed that essentially the same proportion of both assemblies returned a BLASTx hit against the swiss-prot database (75.28% versus 75.22%, Table 7). Nevertheless, 250 more unique database entries were detected in our concatenated transcriptome (Table 7). These two assemblies shared 13,938 entries, while our assembly possessed 2534 unique entries and the Nakasugi et al assembly possessed 2284 unique entries (Table 7). This picture was supported by the BUSCO analysis: both assemblies shared 929 BUSCOs entries (a total of 14 BUSCOs entries were missing in both assemblies suggesting this dataset is largely complete), with five entries unique to our assembly and eight unique to the Nakasugi assembly. In addition, the number of duplicated copies was lower in our assembly than in the assembly reported by Nakasugi et al. (745 versus 785 respectively).

**Table 7:** Comparison of BLASTx annotation rate of both *N. benthamiana* cumulative transcriptomes

	Our study	Nakasugi et al
Total number of transcripts	127,526	234,526
Number of transcripts with BLASTx hits against Swiss-prot	95,929 (75%)	176,540 (75%)
Number of transcripts with a unique Swiss-prot hit	16,472	16,222
Number of shared transcripts with a unique Swiss-prot hit	13,938	
Number unique transcripts with a unique Swiss-prot hit	2,534	2,284

## Conclusion

As far as we are aware this is the first study to characterize the effects of combining multiple *de novo* transcriptome assemblies in order to both maximize the information content, and minimize the redundancy of the resulting coding transcriptome for a variety of eukaryotes. A similar method was previously reported for transcriptomes derived from plants in order to address assembly difficulties associated with polyploidy [10]. Our approach however requires only three alternative assemblies in comparison with many tens of assemblies. In general our methodology produces a more concise and information-rich coding transcriptome assembly that will make subsequent analyses more efficient; from the comparisons we conducted here on six independent eukaryotic datasets using three popular RNA-Seq assembly packages we generated on average 1.8X fewer transcripts, and significantly increased the degree and diversity of annotatability in comparison to any of the three individual assemblers. In addition, we tested our approach on two simulated datasets generated from reference genomes, confirming the results observed from ‘real world’ Illumina datasets. We believe our approach (encoded by the simple perl script provided here) will allow researchers with minimal bioinformatics experience to extract the most information from their RNA-Seq datasets. A weakness we observe in our approach is the generation of slightly more “false” transcripts and redundancy than seen in the individual assemblers we employed. This phenomenon (present in all methods used to assemble RNA-Seq data) will have an impact on subsequent analyses, for example differential gene expression (DGE). In the case of DGE analysis, this weakness can be countered to some extent by allowing multiple read mappings as implemented by Rsubread [29]. This also serves to emphasize the point that such analyses based on NGS data should always be confirmed by independent validation experiments.

## Additional files

Additional file 1: Table S1. Raw NGS data characteristics pre and post quality filtering with Trimmomatic. (XLSX 10 kb)
Additional file 2: Parameter file for reads design with Flux simulator on *D. melanogaster*. (TXT 651 bytes)
Additional file 3: Perl script used to perform our assembly pipeline. (PL 43 kb)
Additional file 4: Perl script used to randomly select candidates from a list of transcripts for our PCR validation experiments. (PL 10 kb)
Additional file 5: Table S2. Assembly metrics of the three sub-assemblies and the final concatenated assembly for each of the six datasets. (XLSX 11 kb)
Additional file 6: Table S3. Clustering metrics for the three sub-assemblies for each of the six datasets. (XLSX 10 kb)
Additional file 7: Table S4. TransDecoder metrics for the concatenated assemblies for each of the six datasets. (XLSX 9 kb)
Additional file 8: Table S5. Effect of the CD-HIT-EST aS value on clustering. (XLSX 13 kb)
Additional file 9: Table S6.
*p*-values generated by Kolmogrov-Smirnov distribution tests. (XLSX 9 kb)

## Abbreviations

BUSCO: Benchmarking Universal Single Copy Orthologs
CDS: Coding DNA sequence
DGE: Differential gene expression
DNA: DeoxyriboNucleic Acid
mRNA: messenger RNA
NGS: Next generation sequencing
ORFs: Open reading frames
PCR: Polymerase chain reaction
RNA: RiboNucleic Acid
SRA: Sequence read archive
